# Efficacy of ruxolitinib cream in a patient with lupus miliaris disseminatus faciei

**DOI:** 10.1016/j.jdcr.2026.04.002

**Published:** 2026-04-09

**Authors:** Emma Chevallier, Nathalie Cardot-Leccia, Thierry Passeron, Henri Montaudié

**Affiliations:** a Department of Dermatology, Hôpital de l’Archet, CHU de Nice, University Côte d’Azur, Nice, France; b Department of Anatomic Pathology, Hôpital de Pasteur, CHU de Nice, University Côte d’Azur, Nice, France; c INSERM U1065, Centre Méditerranéen de Médecin Moléculaire, University Côte d’Azur, Nice, France

**Keywords:** JAK pathway, lupus miliaris, ruxolitinib cream, topical treatment

Lupus miliaris disseminatus faciei (LMDF) is a rare granulomatous inflammatory disease that often manifests on the face as red, brown, or yellow papules. Although benign, LMDF carries a substantial risk of scarring and disfigurement. No standardized treatment has been established; current therapeutic options include oral tetracyclines, metronidazole, dapsone, and isotretinoin with variable efficacy.^1^^,^^2^

Herein, we report the case of a woman in her 60s, with no prior medical history and not taking any medication. She presented with a 1-year history of erythematous papular dermatitis affecting exclusively the upper eyelids (Fig 1, *A*). The cheeks and nose remained unaffected. The lesions were monomorphic reddish papules, without pustules, telangiectasias, or atrophy. They were pruritic, sensitive, occasionally painful, and substantially affected her quality of life (Dermatology Life Quality Index: 16/30).

**Fig 1:**
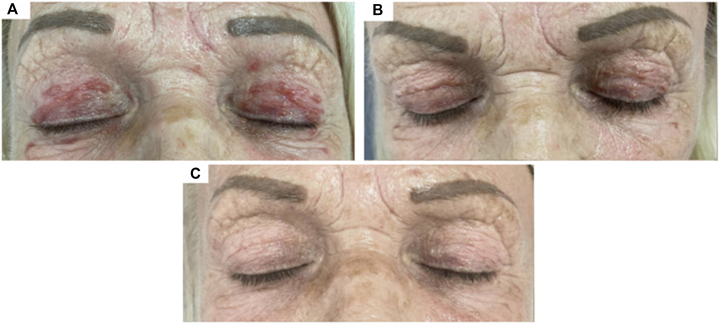
A woman in her 60s with lupus miliaris disseminatus faciei treated with ruxolitinib cream 1.5%. **(A)** Initial presentation before treatment. **(B)** After 2 months of twice-daily applications of ruxolitinib cream. **(C)** After 4 months of treatment.

No ophthalmologic symptoms, flushes, or systemic symptoms were reported. Clinical and dermoscopic examination found no evidence for demodecidosis. No relevant contact allergens or drug exposures were identified. An initial diagnosis of granulomatous rosacea was established by an ophthalmologist, and the patient received first-line treatment with doxycycline (100 mg daily for 2 months, then 200 mg daily for 3 months), without any improvement. Subsequently, she was treated with topical ivermectin 1% (twice daily for 3 months), which also failed to improve her condition, despite good treatment adherence. The patient was referred to our center, where a diagnosis of LMDF was considered. A skin biopsy of 1 eyelid lesion revealed a lymphohistiocytic infiltrate with an epithelioid cell granuloma (Fig 2).

**Fig 2:**
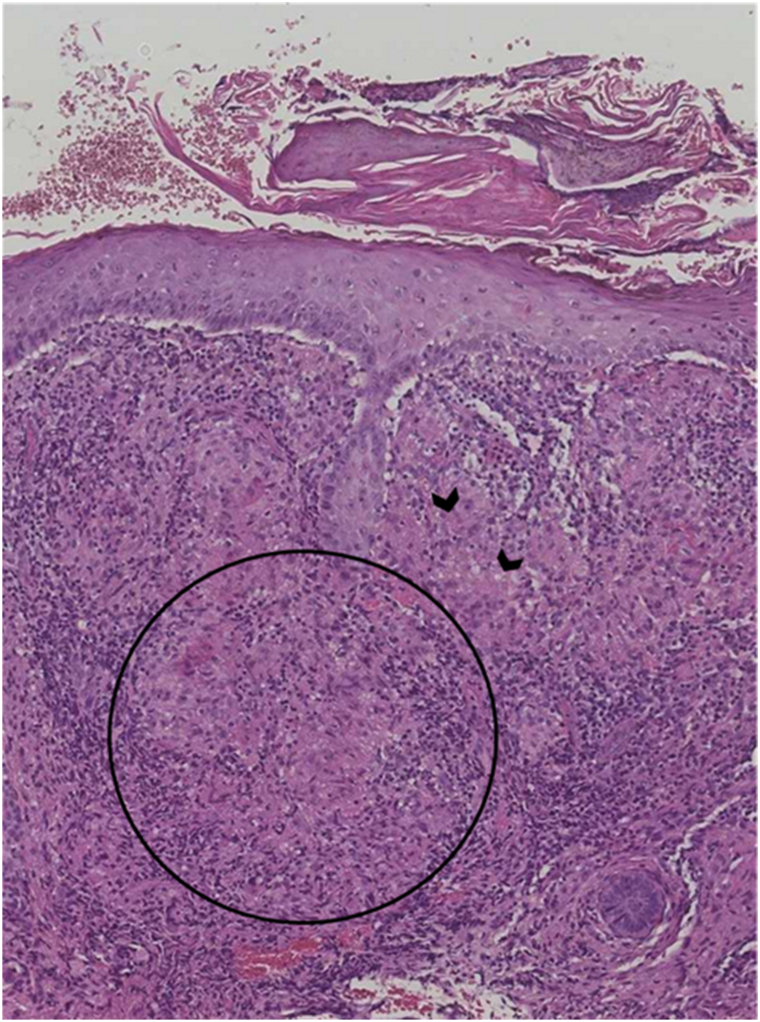
Skin biopsy of a lesion showing an epithelioid cell granuloma without necrosis within a dense lymphohistiocytic infiltrate The black circle indicates the epithelioid cell granuloma. *Arrowheads* indicate the inflammatory infiltrate surrounding the granuloma. (Hematoxylin and eosin stain; original magnification: ×100.)

No demodex was observed. Ziehl-Neelsen, Gram, and Periodic acid–Schiff stains were negative. Polarized light examination did not reveal foreign material. The biological tests carried out were normal, especially the calcium-phosphate profile and angiotensin-converting enzyme levels, excluding sarcoidosis. Based on these data, a diagnosis of LMDF was established. After multidisciplinary discussion, an off-label treatment with ruxolitinib cream 1.5% applied twice daily was initiated. After 2 months of treatment, marked improvement was observed, with a decrease in infiltration and number of eyelid lesions (Fig 1, *B*). At 4 months, the lesions had resolved completely, and the patient reported disappearance of itching and pain, accompanied by a substantial improvement in quality of life (Dermatology Life Quality Index: 1/30, Fig 1, *C*). The treatment was well tolerated, with no adverse effects, especially no herpetic events or ocular complaints. After 6 months of treatment, ruxolitinib cream was discontinued. At 6-month follow-up, without any further treatment, the patient remained in complete remission.

The treatment of LMDF remains challenging and is not well established. Several treatment options have been proposed, most often by analogy with those used for granulomatous rosacea.^1^^,^^3^ Recently, Janus kinase (JAK) inhibitors have been tested for the treatment of various inflammatory skin diseases, including alopecia areata and lichen planus.^4^ Ruxolitinib, a JAK1 and JAK2 inhibitor, inhibits interferon-γ–mediated JAK-signal transducer and activator of transcription signaling.^5^ It is approved for the topical treatment of nonsegmental vitiligo and atopic dermatitis, with good efficacy and safety profile, including use on the eyelids.^6^^,^^7^ Macrophage accumulation, which plays a key role in granuloma formation, is closely linked to the interferon-γ pathway. This partly explains why ruxolitinib is effective in several granulomatous dermatoses.^5^^,^^8^^,^^9^ Gorham et al^10^ reported 1 successfully treated case of LMDF.

Based on these data, the patient’s prior unsuccessful treatments, and to avoid the potential adverse effects of topical corticosteroids on the eyelids, we proposed treatment with ruxolitinib cream 1.5%. In addition to the pathophysiological rationale for its application in the treatment of LMDF,^5^^,^^8^ the potential strong penetration of ruxolitinib through the thin eyelid skin may contribute to its high efficacy. The optimal duration of treatment and the persistence of effectiveness after discontinuation remain to be determined. Although spontaneous remission can occur in LMDF, the rapid and marked efficacy of topical ruxolitinib observed in this severe, treatment-resistant case of LMDF, supports further studies, particularly to evaluate its potential efficacy in granulomatous dermatoses of limited extent.

## Conflict of interest

Dr Montaudié has received honoraria and/or consulting fees from BMS, Pierre Fabre Oncology, MSD, SUNPharma, and REGENERON. Dr Passeron has a consulting and advisory roles with Almirall, AbbVie, Amgen, Bristol-Myers-Squibb, Celgene, Galderma, Incyte, Janssen, Lilly, Léo Pharma, Novartis, Pfizer, Sanofi, SUNPharma, UCB, Vyne Therapeutics and received previous support for travel and accommodation expenses from AbbVie, Incyte, Janssen, Lilly, Novartis, and UCB. Drs Chevallier and Cardot-Leccia have no conflicts of interest to declare.
